# Financial Fraud Identification Based on Stacking Ensemble Learning Algorithm: Introducing MD&A Text Information

**DOI:** 10.1155/2022/1780834

**Published:** 2022-09-20

**Authors:** Zhiheng Zhang, Yong Ma, Yongjun Hua

**Affiliations:** ^1^School of Accounting, Chongqing University of Technology, Banan 400054, Chongqing, China; ^2^School of Economics and Business Administration, Chongqing University, Shapingba 400044, Chongqing, China

## Abstract

In recent years, there have been frequent incidents of financial fraud committed through various means. How to more efficiently identify financial fraud and maintain capital market order is a problem that scholars from all walks of life are discussing and urgently seeking to resolve. In this study, a financial fraud identification model is constructed based on the stacking ensemble learning algorithm, and the text of the management discussion and analysis (MD&A) chapter in annual reports is introduced based on financial and nonfinancial variables, using sentiment polarity, emotional tone, and text readability as text variables. The results show that when considering financial and nonfinancial variables and introducing text variables, the recognition effect of the stacking ensemble learning model constructed in this study is significantly better than the classification results of each single classifier model. In addition, the model recognition effect is better after adding text variables. Therefore, the model is expected to provide a new and more effective method of identifying financial fraud.

## 1. Introduction

Financial fraud is any intentional or deliberate act to deprive another of property or money by guile, deception, or other unfair means [1–3]. Although it has been two decades since the famous Enron scandal in the history of financial fraud, many business managers still choose to take risks as the large benefits brought about by fraud far outweigh the costs. For example, the Kangmei Pharmaceutical Incident and the Luckin Coffee Incident that have occurred in China in recent years have caused sensations throughout the country. As of 2019, Luckin Coffee's directly operated stores had surpassed Starbucks to become the largest chain coffee brand in China. However, on February 1, 2020, the research firm Muddy Waters released an 89-page short-selling report on Luckin Coffee, pointing out that Luckin Coffee began to fabricate financial data and fictitious operating profits in the third quarter of 2019. On April 2, 2020, Luckin Coffee admitted to financial fraud, causing its market value to evaporate by 35.4 billion yuan overnight. Five days later, on April 7, Luckin Coffee's operations were suspended. The financial fraud damaged social interests to a large extent and disrupted the order of the socialist capital market [4, 5]. In this regard, many scholars have invested considerable time and energy to identify financial fraud more effectively and have performed in-depth research in this area.

Machine-learning algorithms can effectively identify financial fraud [6–10]. Some scholars have found that integrated algorithms such as random forests exhibit better identification effects through a comparative analysis of different algorithms [11–13]. However, no financial fraud identification model has been built based on the stacking ensemble learning algorithm. In this study, a financial fraud identification model is constructed based on the stacking ensemble learning algorithm. Three single-classifier models with better classification effects are selected as base learners, and the ensemble model is obtained by fusing multiple single-classifier models through the meta-learner, which provides a new option for more efficient identification of financial fraud to a certain extent.

Many scholars have found that financial indicators can effectively distinguish between fraud and nonfraud [14, 15]. However, only a few scholars have considered textual information when studying financial fraud identification [16]. In this study, in addition to considering financial and nonfinancial indicators, management discussion and analysis (MD&A) text is introduced, and the information that helps improve the identification of financial fraud is extracted from the text through text analysis technology. Combined with financial data, a more comprehensive indicator system is formed. To a certain extent, the financial fraud identification indicator system is enriched.

Furthermore, this study selects as its research sample 171 listed companies punished by the China Securities Regulatory Commission from 2016 to 2020 as fraud samples and 171 nonfraud matching sample companies determined following the one-to-one principle. Taking financial, nonfinancial, and text indicators as sample features, a financial fraud identification model is constructed based on the stacking ensemble learning algorithm. This is expected to provide a new and more effective method for identifying financial fraud.

**Figure 1:**
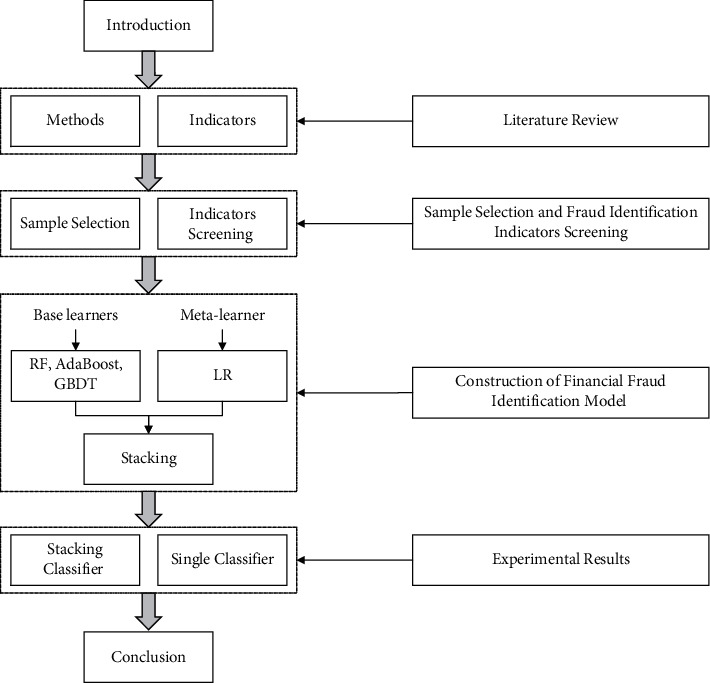
Organizational framework of the paper.

The organizational framework of the paper is summarized in Figure 1. The introduction presents the scope of the study, its motivation, and its contributions.  Section 2 reviews the literature on financial fraud identification models and indicator selection.  Section 3 presents the selection of the research samples, the selection of the indicators, and the indicator screening process. The financial fraud identification model building process is discussed in Section 4.  Section 5 presents the experimental results. The conclusions and limitations of the study are presented in Section 6.

## 2. Literature Review

At present, research on financial fraud identification in academia mainly starts from two aspects. The first is research on financial fraud identification methods. The second is the study of financial fraud identification indicators.

### 2.1. Research on Financial Fraud Identification Methods

Early on, some scholars used the *M*-Score [17–19], *F*-Score [20], and *Z*-Score [21] models to evaluate the possibility of financial fraud. Some scholars have also applied Benford's law to the identification of financial fraud in the accounting field based on the distribution law of the first digit in the dataset and verified its applicability [22].

In recent years, with the rapid development of artificial intelligence technology, machine learning (ML) algorithms such as logistic regression (LR) [23], back propagation neural networks (BPNNs) [24], support vector machines (SVMs) [25], and decision trees (DTs) [26] have been employed in the field of financial fraud identification by many scholars. Various deep-learning algorithms have also been employed in financial fraud identification research. Examples include convolutional neural networks (CNNs) [27], long short-term memory (LSTM) [28], hierarchical self-attention (HSA) [29], and self-organizing maps (SOMs) [30]. However, single-classifier models are limited by the models themselves, and their performance improvement has become a bottleneck; hence, many scholars have turned their attention to ensemble-learning algorithms.

At present, more mature ensemble learning algorithms mainly include the bagging, boosting, and stacking algorithms. Some scholars have also proposed hybrid prediction models [31, 32]. The bagging algorithm performs random sampling of the dataset, eventually forming multiple single classifiers, and obtains the final classification result through the voting method; that is, the minority obeys the majority. The typical representative of the bagging algorithm is the random forest (RF) algorithm based on the DT. Ye et al. detected financial statement fraud using the RF algorithm, and the results showed that the RF model was superior to single-classifier models such as LR, SVMs, and DTs [33]. Later, An and Suh established a financial statement fraud identification model that exhibited better classification performance using an improved RF algorithm [34]. The boosting algorithm is similar to the bagging algorithm; the difference is that the single classifiers in the bagging algorithm are parallel, whereas the single classifiers in the boosting algorithm are performed in an iterative manner, which is serial. At the same time, different weights are given to every classifier to reduce the training errors to a greater extent and improve the recognition accuracy. Boosting algorithms mainly include adaptive boosting (AdaBoost) [35, 36], gradient boosting decision tree (GBDT) [37], and extreme gradient boosting (XGBoost) [38]. However, both bagging and boosting algorithms integrate single classifiers through simple voting or weighted voting, whereas the stacking algorithm combines single classifiers through a new learner. Pisula used a stacking ensemble model to predict the bankruptcy risk of Polish companies [39]. Liang et al. also constructed a bankruptcy prediction model based on the stacking ensemble model and demonstrated that the stacking ensemble algorithm had a better recognition effect [40]. This is sufficient to illustrate the applicability of the stacking ensemble learning model in the field of accounting research and provides experience for the application of the stacking ensemble model in financial fraud identification.

### 2.2. Study of Financial Fraud Identification Indicators

Many scholars have always selected financial ratio indicators [41, 42] as fraud risk identification indicators when studying financial fraud identification, and some scholars have included nonfinancial indicators such as ownership structure [28] in the model indicator input. Few scholars have considered introducing textual information in annual reports into the indicators, and only a few scholars have attempted to do so. Goel and Uzuner proposed a method to analyze the qualitative content of annual reports, using natural language processing (NLP) technology to analyze the MD&A text content in annual reports from the dimensions of sentiment polarity. The findings suggest that the financial reports of fraudulent firms contain higher emotional content [43]. Craja et al. used a combination of financial ratios and textual information in a company's annual report to detect financial statement fraud when studying the identification of financial reporting fraud. Extracting text features from MD&A in annual reports as incremental information for financial ratios, research results show that this improves the recognition accuracy of the model to a certain extent [44]. Dong and Liu also used deep learning and NLP technology to propose a domain-adaptive financial text sentiment classification method that predicts financial crises by classifying financial texts. Their results showed that classification accuracy was significantly improved [45]. This is sufficient to show that text information helps improve the identification of financial fraud. Therefore, this study introduces MD&A text information in the identification of financial fraud to explore the extent to which text information improves classification accuracy.

It can be seen from the above that a large number of scholars have devoted themselves to the study of financial fraud identification and have achieved fruitful research results. Many attempts have been made in the selection of recognition methods, whether it is a single-classifier model or an ensemble learning model. Many scholars have found that ensemble learning models are better than single-classifier models by comparing the recognition effects of different models. However, the ensemble learning models used by many scholars at present integrate every single classifier through simple or weighted voting. The stacking ensemble learning algorithm integrates every classifier through a learner, which has a better recognition effect. In the selection of financial fraud identification indicators, most scholars only consider financial and nonfinancial variables and do not consider the text information in annual reports. However, many scholars have shown that there is much valuable information contained in the annual report text, which helps improve the identification of financial fraud. Therefore, based on the stacking ensemble learning algorithm, this study selects the RF, AdaBoost, and GBDT algorithms with better classification effects as the base learners of the model and selects the LR algorithm as the meta-learner to construct a financial fraud identification model. The models are compared and analyzed to test whether the recognition effect of this study's financial fraud recognition model is better than that of single-classifier models. In financial fraud identification indicators, MD&A text information is introduced as incremental information of financial and nonfinancial variables, and sentiment polarity, emotional tone, and text readability indicators are used as text variables to explore whether MD&A text can be used as incremental information of financial data and more effectively identify corporate financial fraud.

## 3. Sample Selection and Fraud Identification Indicators Screening

### 3.1. Sample Selection

Before 2015, the MD&A texts of some listed companies had not been disclosed separately in their annual reports; hence, the data selection range in this study is from 2016 to 2020. Based on the China Securities Regulatory Commission, the Shanghai Stock Exchange, and the Shenzhen Stock Exchange, we select listed companies that were first punished for fictitious profits, false records, fictitious assets, and major omissions as fraud samples. Using the one-to-one matching principle, nonfraud samples in the same industry and year are strictly selected as control samples; the label of fraud samples is recorded as 1, and the label of nonfraud samples is recorded as 0. A total of 171 fraudulent and 171 nonfraudulent samples are screened.

The financial and nonfinancial data in this study are from the China Stock Market Accounting Research (CSMAR) database, which includes databases on violation handling, financial indicator analysis, and governance structure. Textual data were sourced from the Management Discussion and Analysis Database of the Chinese Research Data Services Platform (CNRDS).

### 3.2. Selection of Financial Fraud Identification Indicators

#### 3.2.1. Financial and Nonfinancial Indicators

Financial indicators are calculated based on financial statements. Some scholars have found that abnormal financial indicator data can provide direct clues for the identification of financial fraud [46, 47]. In this regard, in accordance with the principles of scientific, systematic, and comprehensive variable selection, this study refers to relevant research on the identification of financial fraud by Zheng et al. [48], selecting financial indicators from eight aspects: solvency, operating capacity, profitability development ability, per share index, cash flow index, relative value index, and risk level. Among these, solvency reflects the ability of an enterprise to repay its debts. Operational capacity reflects an enterprise's ability to operate its assets. Profitability reflects an enterprise's ability to use capital to obtain profits. Development ability reflects changes in accounting elements, such as enterprise assets, in the vertical comparison. The per share index mainly reflects a company's operating results and can measure the level of stock profitability and investment risk. The cash flow index reflects the cash flow generated by a company's investments, financing, and operating activities. If a company uses a related party for fictitious transactions or false invoices, it can be directly reflected in the cash flow. The relative value index can also reflect the differences in the indicators between fraudulent and nonfraudulent companies. Financial and operating leverage reflect the risk level of an enterprise, and a higher risk level affects the financing ability of the enterprise. In addition, ownership concentration, the concurrent positions of chairman and general manager, the proportion of tradable shares, the proportion of independent directors, and the type of audit opinion nonfinancial indicators were also selected to obtain preliminary financial and nonfinancial indicators. Ownership concentration is the sum of the shareholding ratios of the top ten shareholders. The concurrent positions of chairman and general manager refer to whether the two positions are held by the same person; if so, it is recorded as 1; if not, 0. The proportion of tradable shares is the number of outstanding shares of the company divided by the total share capital. The proportion of independent directors refers to the proportion of independent directors on the board of directors of an enterprise. The type of audit opinion refers to the audit opinion issued by the accounting firm to the enterprise; a standard audit opinion is recorded as 0, and a nonstandard audit opinion is recorded as 1. The financial and nonfinancial indicator systems are initially constructed from nine aspects, including solvency, operating capacity, profitability, development ability, per share index, cash flow index, relative value index, risk level, and nonfinancial indicator, as shown in Table 1.

#### 3.2.2. Analysis of Text and Calculation of Indicators

Textual analysis refers to the process of converting unstructured text information into structured data that can be represented by numerical values. Limited by the technical level in the early days of research, there has been almost no research related to text analysis. With the rapid development of artificial intelligence technology, many scholars have conducted research on different text analysis methods in recent years. Current text analysis techniques have mainly included topic analysis, dictionary, bag-of-words (BOW), ML, and NLP methods. In the financial field, the BOW and ML methods are mainly used. The BOW method treats text as a collection of a large number of words, and the text is vectorized by calculating the word frequency. The ML method determines the classification rules using pretrained models and then applies them for text analysis. Choi et al. used the BOW method, word-to-vector (Word2Vec), and document-to-vector (Doc2Vec) three text analysis techniques to analyze a MD&A corpus of financial reports, and the results showed that BOW mostly outperforms Word2Vec and Doc2Vec [49]. Therefore, this study selects the BOW method to analyze MD&A text information in financial reports.

However, the premise of using the BOW method well is to have a mature dictionary, especially for a specific field. The LM financial sentiment dictionary created by Loughran and McDonald is widely used in text analysis in the field of accounting [50]. Many Chinese scholars have also used the LM sentiment dictionary as a vocabulary list for text analysis. However, word usage between Chinese and English differs largely in many ways, and it remains doubtful whether simply translating an English dictionary into Chinese provides accurate insights. Therefore, some Chinese scholars have drawn on the LM financial sentiment dictionary and combined it with the CNKI thesaurus, the Dalian University of Technology sentiment thesaurus, the Tsinghua University praise and derogation dictionary, and an annual report corpus to construct a Chinese sentiment dictionary in the financial field. This study draws on the Chinese sentiment dictionary for the text analysis process.

The MD&A text in the financial reports of listed companies is an effective supplement to statement data, which contains much valuable information and can be used as incremental information to identify financial fraud. Studies have shown that, compared with nonfraud companies, the management of fraudulent companies is more likely to use positive words to describe company operations and prospects, and fraudulent companies prefer to use long sentences or highly specialized sentences to reduce text readability [51]. On this basis, this study draws on the research of Lo et al. [52] and Loughran and McDonald [53]. Positive and negative are chosen as sentiment polarity indicators. Strong and weak modalities are used as emotional tone indicators. Text readability indicators include professional terms, average sentence length, and text length. We quantify text metrics as the ratio of the frequency of words in the thesaurus of various metrics to the total number of words in the MD&A text, as listed in Table 2.

**Figure 2:**
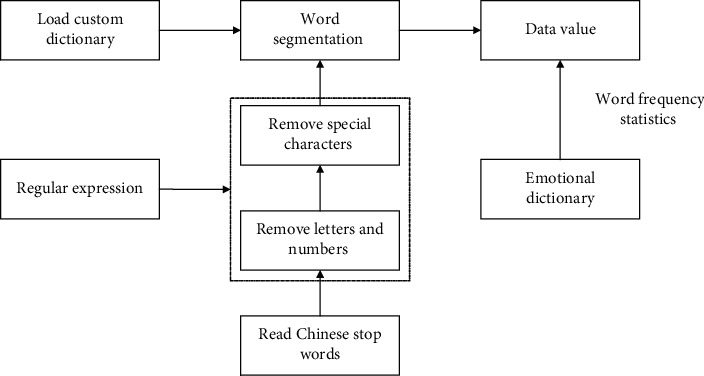
Schematic diagram of text analysis and index calculation process.

This study converts text to numeric values according to the steps shown in Figure 2. The entire text analysis process is completed using Python programming tools. The third-party libraries used are jieba, pandas, and re. The jieba tool is used to perform word segmentation on the MD&A text. The pandas tool is used to read and output the data. The re tool is mainly used to regularize text to facilitate word segmentation. The first step in text analysis is to load a custom dictionary to ensure that the word segmentation results meet one's requirements. For example, after the word *financial statement* is added to the custom dictionary, it is not divided into two words (*financial* and *statement*). The second step is to segment the MD&A text. First, Chinese stop words are read and meaningless words such as modal particles are removed. Then, regular expressions are used to remove numbers and special characters from the MD&A text, which helps with word segmentation. Then, a word segmentation process is carried out for the MD&A text. The last step is based on different sentiment dictionaries, counting the frequency of words and obtaining the output value. The Chinese sentiment dictionary is used to analyze text sentiment polarity, including positive and negative; the CNKI sentiment dictionary is used to analyze the emotional tone of texts, including strong modal and weak modal; and the Sogou cell thesaurus is used to analyze the professional terms in the text. Finally, dividing the word frequency of each indicator by the total number of words in the MD&A is the index value.

### 3.3. Screening of Financial Fraud Identification Indicators

Through a preliminary selection of variables, this study selects 81 financial indicators and five nonfinancial indicators from nine aspects, analyzes the MD&A text information in annual reports, and selects seven text indicators to construct a comprehensive coverage financial fraud identification indicator system. However, too many indicators are prone to problems such as the strong correlation between indicators, resulting in overfitting of the model, resulting in a small training error of the model and a large test error, resulting in poor generalization ability of the model and affecting model performance. Therefore, this study performs principal component analysis (PCA) on indicators to reduce the dimension of the indicator input. The principal component calculation formula is as follows:

(1) $$\begin{array}{c} F=RX=\left(\begin{array}{ccc} \lambda_{11}X_{1} & \cdots & \lambda_{1n}X_{n}\\ \vdots & \ddots & \vdots \\ \lambda_{k1}X_{1} & \cdots & \lambda_{kn}X_{n} \end{array}\right) \end{array}$$

In the formula, *X* represents the original indicator, *R* represents the eigendirection of the top *k* largest eigenvalues in the covariance matrix of *X*, and F represents the principal component vector.

This study uses SPSS 22 to perform a PCA on the data. First, KMO and Bartlett's sphericity tests are performed to test whether the data are suitable for factor analysis. The results show that the KMO value of 81 financial indicators is 0.732, and the significance is 0.000 < 0.05, which is suitable for factor analysis. The KMO value of five nonfinancial indicators is 0.5, which is not suitable for a factor analysis; hence, the five nonfinancial indicators are all used as indicator inputs. Second, the PCA of the financial indicators is performed, and the results are shown in Table 3. It can be seen from the table that a total of 23 components have eigenvalues greater than 1, and their cumulative variance contribution rate is 78.449%, indicating that these 23 principal components have good representativeness and explanatory power and that the factor analysis results are ideal. Therefore, this study uses these 23 principal components instead of financial indicators as inputs and denotes these as *F*1, *F*2,…, *F*23.

Therefore, after dimensionality reduction of the variables, 28 financial and nonfinancial indicators and seven text indicators are reserved as the final indicator input of the financial fraud identification model.

### 3.4. Normalization of Data

Since this study's selected indicators have different dimensions and units, to avoid the influence of data of different magnitudes on the model, this study uses the maximum and minimum normalization methods to normalize all financial indicator data. Finally, all data values are scaled to the interval [0, 1]. The conversion formula is as follows:

(2) $$\begin{array}{c} x^{\prime }=\frac{x-x_{\min}}{x_{\max}-x_{\min}.} \end{array}$$

## 4. Construction of the Financial Fraud Identification Model

This section builds the financial fraud identification model based on stacking ensemble learning and expounds on the algorithm principle of its base learners and meta-learner. The stacking ensemble learning algorithm can integrate different classifiers. The basic idea is to first train multiple different base learners through cross-validation, then combine the outputs of each base learner as the training dataset of the meta-learner, and then perform secondary training combined with the data labels to obtain the final output result.

Based on the stacking ensemble learning algorithm, this study selects RF, AdaBoost, and GBDT as the base learners, selects the LR algorithm as the meta-learner, and builds the financial fraud identification model based on the stacking ensemble learning algorithm.

### 4.1. Base Learner Algorithm

#### 4.1.1. Random Forest Algorithm

**Figure 3:**
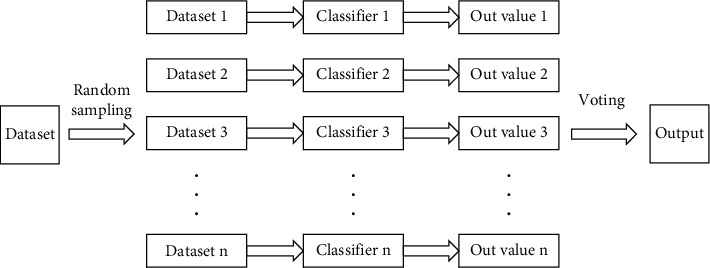
RF algorithm schematic diagram.

The random forest algorithm is a typical representative of the bagging algorithm. The basic principle is to perform random sampling *n* times on the training dataset, which includes both the randomness of sample selection and the randomness of indicator selection, to obtain *n* different single classifiers based on a classification decision tree model. The final output result is obtained by voting according to the recognition results of each classifier. The principle of the RF algorithm is shown in Figure 3.

#### 4.1.2. Adaptive Boosting Algorithm

**Figure 4:**
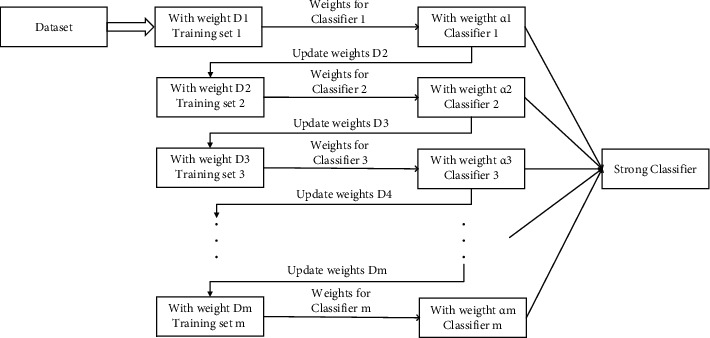
AdaBoost algorithm schematic diagram.

The adaptive boosting algorithm is a typical representative of the boosting algorithm. Its basic principle is to assign certain weights to the training samples, form multiple single classifiers through iterations, and gradually update the sample weights. Finally, the weighted summation of each model is used to obtain the final output. The principle of the AdaBoost algorithm is shown in Figure 4.

#### 4.1.3. Gradient Boosting Decision Tree Algorithm

The gradient boosting decision tree algorithm is also a boosting algorithm. The core of the algorithm is to use the residual of the previous learner to fit a new learner, generate multiple single classifiers through iterations, and finally combine them into a strong classifier through an additional model. The GBDT binary classification algorithm process is as follows:

Step 1: initialize the first classifier:
  (3) $$\begin{array}{c} F_{0}\left(x\right)=\log\frac{P\left(Y=1|X\right)}{1-P\left(Y=1|X\right)}\text{.} \end{array}$$
In the formula, *P*(*Y*=1*|X*) represents the probability that the sample label in the training dataset is 1.
Step 2: establish *M* classification and regression trees, *m*=1,2 ⋯ , *M*:
  (a) Calculate the negative gradient, that is, the residual, *i*=1,2 ⋯ , *N*:
    (4) $$\begin{array}{c} r_{mi}=-{\left[\frac{\partial L\left(y_{i},F\left(x_{i}\right)\right)}{\partial F\left(x\right)}\right]}_{F\left(x\right)=F_{m-1}\left(x\right)}=y_{i}-\frac{1}{1+\exp\;\left(-F\left(x_{i}\right)\right)} \end{array}$$
  (b) Update the sample label value of the residuals calculated in the previous step, use (*x*_*i*_, *r*_*mi*_) as a new training dataset, and obtain a new classification and regression tree and its corresponding leaf node area through training *R*_*mj*_, *j*=1, 2,⋯, *J*_*m*_, where *J*_*m*_ represents the number of leaf nodes of the *m*th tree.
  (c) Calculate the best fitting value for *J*_*m*_ leaf node regions:
    (5) $$\begin{array}{c} c_{mj}=\frac{\underset{x_{i}\in R_{mj}}{\sum }r_{mj}}{\underset{x_{i}\in R_{mj}}{\sum }\left(y_{i}-r_{mj}\right)\left(1-y_{i}+r_{mj}\right)} \end{array}$$
  (d) Update the learner *F*_*m*_(*x*):
    (6) $$\begin{array}{c} F_{m}\left(x\right)=F_{m-1}\left(x\right)+\overset{J_{m}}{\underset{j=1}{\sum }}c_{mj}I\left(x\in R_{mj}\right)\text{.} \end{array}$$
Step 3: Obtain the final learner *F*_*M*_(*x*):
  (7) $$\begin{array}{c} F_{M}\left(x\right)=F_{0}\left(x\right)+\overset{M}{\underset{m=1}{\sum }}\overset{J_{m}}{\underset{j=1}{\sum }}c_{mj}I\left(x\in R_{mj}\right) \end{array}$$

### 4.2. Meta-Learner Algorithm

#### 4.2.1. Logistic Regression Algorithm

The logistic regression algorithm is often used by many scholars in the study of classification problems owing to its advantages of easy understanding and fast operation. The basic principle of this method for identifying financial fraud is that *k* known sample indicators are input as independent variables, that is, *X* = {*X*_1_, *X*_2_, *X*_3_, ⋯, *X*_*K*_}, and the sample label is used as a dependent variable; that is, *Y* = 1 indicates a fraudulent enterprise, and *Y* = 0 indicates a nonfraudulent enterprise. The logistic model is shown in the following equations:

(8) $$\begin{array}{c} \ln\left(\frac{p}{1-p}\right)=\beta_{0}+\beta_{1}X_{1}+\beta_{2}X_{2}+\cdots +\beta_{k}X_{k} \end{array}$$

Simplifying formula (8), we obtain formula (9).

(9) $$\begin{array}{c} p=\frac{1}{1+\exp\;\left[-\left(\beta_{0}+\beta_{1}X_{1}+\beta_{2}X_{2}+\cdots +\beta_{k}X_{k}\right)\right]} \end{array}$$

In formulas (8) and (9), *p* represents the probability of fraud, 1 − *p* represents the probability of non-fraud, and *β* represents the regression coefficient of *X*, which is obtained through model training. In this study, the model threshold is set to 0.5; that is, when *p* ≥ 0.5, the enterprise is classified as fraudulent, and when *p* < 0.5, it is classified as nonfraudulent.

### 4.3. Financial Fraud Identification Model Based on Stacking Ensemble Learning

Based on the stacking ensemble learning model, this study selects the RF, AdaBoost, and GBDT algorithms as the base learners and selects the LR algorithm with a simple principle as the meta-learner, thus constructing a financial fraud identification model based on stacking ensemble learning. The implementation of this model can be divided into four steps:

  Step 1: divide the dataset into training and test sets at a ratio of 7 : 3.
  Step 2: train the RF, AdaBoost, and GBDT models based on the training dataset and obtain the output results through cross-validation and testing.
  Step 3: the five-fold cross-validation output results are stacked vertically, and the average of the five test output results is taken as the test output result of the model.
  Step 4: horizontally stack the cross-validation output results of the three models, combine the sample labels as a new training set, horizontally stack the test output results, combine the sample labels as a new test set, and obtain the final output through the LR model.

**Figure 5:**
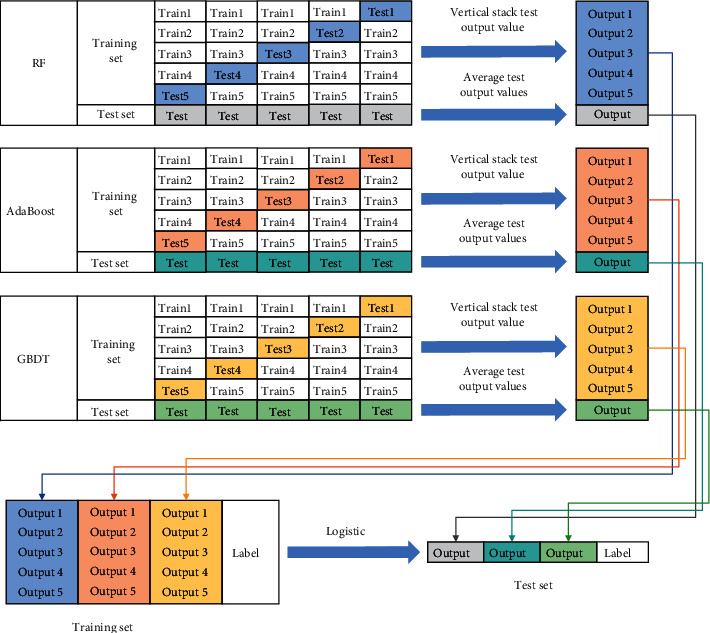
Financial fraud identification model based on stacking ensemble learning.

The principle of the financial fraud identification model based on stacking ensemble learning is illustrated in Figure 5.

## 5. Experimental Results

This section analyzes the identification results of financial fraud. First, the indicators of model evaluation are introduced. Then, the identification results of the financial fraud identification model constructed based on the stacking ensemble learning algorithm are analyzed. Finally, the financial fraud identification model constructed based on the stacking ensemble learning algorithm and the single-classifier model are compared and analyzed. Among these, the financial data are 28 indicators after dimensionality reduction by principal component analysis. Text data are seven indicators after text analysis. The stock code, sample year, and sample label form panel data with financial data and text data, which are the model inputs for financial fraud identification.

**Figure 6:**
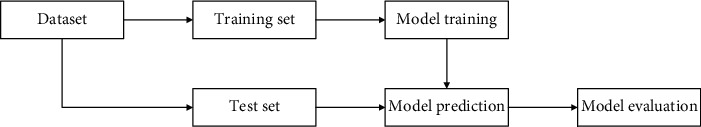
Model implementation process.

The experimental process in this study is based on Python programming tools and completed on the Jupyter platform. The specific implementation steps are shown in Figure 6.

The first step is to divide the dataset into training and test sets according to the ratio of 7 : 3 and separate the data and labels so that the model can use the data to train and predict the label. Then, the segmented training set data are input into the model training, and the test set data are input into the model for prediction. Finally, the predictive effect of the model is evaluated based on the model evaluation indicators.

### 5.1. Evaluation Metrics of the Model

Based on the stacking ensemble learning framework, this study selects the RF, AdaBoost, and GBDT machine learning algorithms as its base learners and the LR algorithm as the meta-learner to construct a financial fraud identification model. The MD&A text information is introduced into the input of the model variable to study whether the text information in the annual reports of listed companies provides incremental information for the identification of financial fraud.

As shown in Table 4, the binary classification problem eventually has four classification cases. These are true positives (TP), false positives (FP), true negatives (TN), and false negatives (FN) [54, 55]. When the model classifies the sample as positive, and it is actually positive, it is called a TP. When the model classifies a sample as positive, but it is actually negative, it is called a FP. When the model classifies a sample as negative, but it is actually positive, it is called a FN. When the model classifies the sample as negative, and it is actually negative, it is called a TN.

For the evaluation of the model, the accuracy of the model classification is mostly used as a measure. On this basis, combined with the confusion matrix, this study uses accuracy, precision, recall, *F*1-score, and area under curve (AUC) values to evaluate the model. The evaluation index is as follows:

(a) Accuracy refers to the probability that the model correctly classifies samples according to the given label. The closer the value is to 1, the better is the model effect.
  (10) $$\begin{array}{c} Accuracy=\frac{TP+TN}{TP+FP+TN+FN} \end{array}$$
(b) Precision, which represents the proportion of samples classified as fraudulent that are actually fraudulent.
  (11) $$\begin{array}{c} Precision=\frac{TP}{TP+FP} \end{array}$$
(c) Recall rate, which represents the proportion of all fraudulent samples that are correctly classified.
  (12) $$\begin{array}{c} Recall=\frac{TP}{TP+FN} \end{array}$$
(d) The *F*1-score is the harmonic mean of precision and recall. The closer the value is to 1, the better is the model effect.
  (13) $$\begin{array}{c} F1-score=\frac{2\text{PR}}{P+R}\text{.} \end{array}$$
(e) The AUC value represents the area enclosed by the ROC curve with the false positive rate (FPR) as the horizontal axis, the true positive rate (TPR) as the vertical axis, and the area enclosed by the horizontal axis of the coordinate axis. Its value range is between [0.5, 1]. The closer the value is to 1, the better is the model effect.
  (14) $$\begin{array}{c} FPR=1-P,TPR=1-R \end{array}$$

### 5.2. Analysis of Identification Results Based on the Stacking Ensemble Learning Model

#### 5.2.1. Comparison of Identification Results by Introduced Text Information

To verify whether the text information in the financial reports of listed companies provides incremental information for improving the effectiveness of financial fraud identification, this study builds a financial fraud identification model based on stacking ensemble learning, respectively considering only financial and nonfinancial variables (*F*) and introducing text variables (*T*). The results are compared and analyzed, and the classification results are presented in Table 5.

The empirical results show that the recognition accuracy of the model after adding text variables reaches 0.8738, which is better than 0.8447 when only financial variables are considered (an increase of 0.0291). Its recall rate has the most obvious improvement effect, which is 0.0724 higher than when only considering financial variables, which means that more fraud samples are correctly identified by the model after adding text variables. This explains that MD&A text information in financial reports provides incremental information for financial fraud identification.

#### 5.2.2. Influence of Each Text Indicator on the Identification Results

Through empirical testing, it has been proven that the introduction of text variables can improve the recognition ability of the model. To further test which type of text indicator has a more significant impact on the model recognition results, this study sets up three groups of experiments based on financial and nonfinancial variables (*F*), adding sentiment polarity, emotional tone, and text readability to the text variables. The results are shown in Table 6.

Further experimental results show that after adding the sentiment polarity index and text readability indicator to the text variables, respectively, the recognition effect of the model is improved compared with only considering financial and nonfinancial variables, and adding text readability after the index, the recognition results of the model are improved more clearly, the recognition accuracy rate reaches 0.8641, and the AUC value reaches 0.8604.

### 5.3. Comparative Analysis with a Single-Classifier Model

To verify whether the performance of the financial fraud identification model based on the stacking ensemble learning algorithm is improved compared with a single classifier model, the financial and nonfinancial variables and the introduction of text variables are considered separately. The financial fraud identification model constructed in this study is compared with the identification results of the RF, AdaBoost, and GBDT models.

#### 5.3.1. Analysis Based on Financial and Nonfinancial Variables

**Figure 7:**
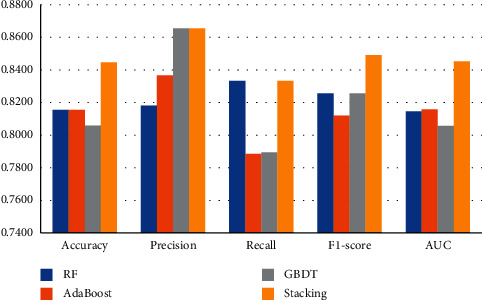
Analysis based on financial and nonfinancial variables.

As shown in Table 7 and Figure 7, when only financial and nonfinancial variables are considered, the overall classification effect of the stacking ensemble model is better than that of the other single-classifier models. From the results of various indicators, the stacking model has an average recognition accuracy rate of 0.0357 higher than other single-classifier models, an average precision of 0.0253 higher, an average recall rate of 0.0295 higher, an average *F*1 value of 0.0279 higher, and an average AUC value of 0.0364 higher. It is verified that the financial fraud identification model constructed based on stacking ensemble learning has a better identification performance than a single classifier.

#### 5.3.2. Introduction of Text Variables

**Figure 8:**
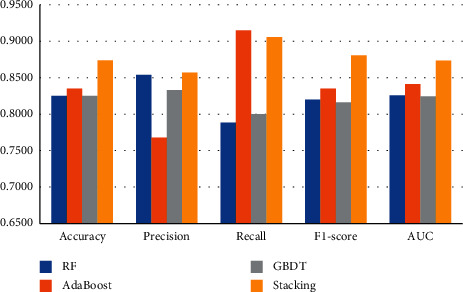
Introduction of text variables.

As Table 8 and Figure 8 show, after the introduction of text variables, the overall classification effect of the ensemble model constructed in this study is still better than that of a single classifier model. Except for the recall rate, which is slightly lower than that of the AdaBoost model, the other indicators are higher than those of the single-classifier model. Among them, the recognition accuracy is 0.0453 higher on average than the single classifier, the precision is 0.0387 higher on average, the recall rate is 0.0712 higher on average, the *F*1-score is 0.0569 higher on average, and the AUC value is 0.0428 higher on average. It also verifies that the recognition effect of the financial fraud recognition model constructed in this study is better than that of a single-classifier model.

It can be seen that whether based on financial and nonfinancial variables or the introduction of text variables, the recognition effect of a financial fraud identification model based on the stacking ensemble learning algorithm is higher than that of other single-classifier models, and the overall effect is better after the introduction of text variables. Additionally, after adding text variables to each single classifier model, the model recognition performance is also improved to a certain extent. After adding the text variable, the recognition accuracy increases by 0.0162 on average, the recall rate increases by 0.0307 on average, the *F*1-score increases by 0.0027 on average, and the AUC value increases by 0.0185 on average. Although the accuracy is reduced by 0.0217 on average, the overall performance of the model has been improved, which further verifies that adding text variables can improve the recognition effect of the model.

## 6. Conclusion

This study builds a financial fraud identification model based on the stacking ensemble learning algorithm and considers one-to-one matching fraud and nonfraud samples from 2016 to 2020 as the research objects. In terms of variable selection, in addition to traditional financial and nonfinancial variables, text variables are also introduced, including sentiment polarity, emotional tone, and text readability, to verify whether textual information provides incremental information for improving financial fraud identification. The research conclusions are as follows: First, after the introduction of MD&A text information, the recognition accuracy of this study's financial fraud model reaches 0.8738, an increase of 0.0291 compared with only considering financial variables. The recognition accuracy of each single classifier model also increases by an average of 0.0162 after adding the text variable, which proves that the text variable improves the recognition quality of the model. This shows that the MD&A text information in corporate annual reports provides incremental information for identifying financial fraud and can more effectively identify corporate financial fraud. Second, among the text variables, both the text readability and sentiment polarity indicators improve the model recognition effect, and the gain information brought about by the text readability indicator is higher. After adding the text readability indicator to the financial variables, the accuracy rate of this study's financial fraud identification model reaches 0.8641, which is 0.0194 higher than that when only considering financial variables. Third, compared with the single classifier model, this study's financial fraud identification model constructed based on stacking ensemble learning exhibits a better recognition effect. When considering financial and nonfinancial variables and introducing text variables, the recognition accuracy increases by 0.0357 and 0.0453, respectively, compared with the single classifier model.

The limitation of this study is that the base learners of stacking ensemble learning encompassed the selection of only three algorithms: RF, AdaBoost, and GBDT. Several other single-classifier algorithms should be considered in future research to discuss the impact of different single classifiers on the stacking ensemble learning algorithm.

## Figures and Tables

**Table 1:** Primary financial fraud identification indicators (financial and nonfinancial indicators).

Dimension	Indicator name
*A* solvency	*A*1 current ratio; *A*2 quick ratio; *A*3 cash ratio; *A*4 equity ratio; *A*5 equity multiplier; *A*6 asset-liability ratio; *A*7 interest protection multiples; *A*8 tangible asset-liability ratio; *A*9 rate of long-term capital accumulation

*B* operating capacity	*B*1 inventory turnover; *B*2 receivables turnover ratio; *B*3 current assets turnover; *B*4 noncurrent assets turnover; *B*5 fixed assets turnover; *B*6 total assets turnover; *B*7 shareholders' equity turnover; *B*8 working capital turnover; *B*9 accounts payable turnover; *B*10 cash turnover; *B*11 capital intensity; *B*12 operating cycle

*C* profitability	*C*1 net interest rate of total assets; *C*2 net interest rate of current assets; *C*3 net interest rate of fixed assets; *C*4 return on equity; *C*5 return on assets; *C*6 operating gross margin; *C*7 operating margin; *C*8 net operating profit margin; *C*9 total operating expenses ratio; *C*10 period expense ratio; *C*11 ratio of profits to cost; *C*12 rate of investment income; *C*13 return on investment; *C*14 return on long-term capital

*D* development ability	*D*1 rate of capital accumulation; *D*2 total assets growth rate; *D*3 increasing rate of fixed assets; *D*4 growth rate of return on equity; *D*5 growth rate of net profit; *D*6 growth rate of operating margin; *D*7 growth rate of total profit; *D*8 growth rate of operating income; *D*9 operating cost growth rate; *D*10 sustainable growth rate; *D*11 owner's equity growth rate

*E* per share index	*E*1 earnings per share; *E*2 total operating income per share; *E*3 earnings per share before interest and tax; *E*4 operating profit per share; *E*5 net assets per share; *E*6 tangible assets per share; *E*7 liabilities per share; *E*8 capital reserve per share; *E*9 surplus reserve per share; *E*10 undistributed profit per share; *E*11 cash flow per share from operating activities; *E*12 cash flow per share from investing activities; *E*13 cash flow per share from financing activities; *E*14 corporate free cash per share flow; *E*15 depreciation and amortization per share

*F* cash flow index	*F*1 net cash content of net profit; *F*2 net cash content of operating income; *F*3 net cash content of operating profit; *F*4 net cash flow of creditors in financing activities; *F*5 net cash flow of shareholders in financing activities; *F*6 total cash recovery rate; *F*7 operating index; *F*8 cash suitable ratio; *F*9 cash reinvestment ratio

*G* relative value index	*G*1 price earnings ratio; *G*2 price book ratio; *G*3 price sales ratio; *G*4 market capitalization tangible asset ratio; *G*5 Tobin's Q value; *G*6 book-to-market value ratio; *G*7 common stock interest rate; *G*8 earnings before interest, tax, depreciation, and amortization

*H* risk level	*H*1 financial leverage; *H*2 operating leverage; *H*3 joint leverage

*I* nonfinancial indicator	*I*1 ownership concentration; *I*2 concurrent positions of chairman and general manager; *I*3 proportion of tradable shares; *I*4 proportion of independent directors; *I*5 type of audit opinion

**Table 2:** Primary financial fraud identification indicators (text indicators).

Dimension	Indicator	Definition
Sentiment polarity	Positive	Positive words/MD&A words
	Negative	Negative words/MD&A words

Emotional tone	Strong modal	Strong modal words/MD&A words
	Weak modal	Weak modal words/MD&A words

Text readability	Professional term	Professional term words/MD&A words
	Average sentence length	Text length/sentence numbers
	Text length	Text length

**Table 3:** Total variance explained.

Component	Initial eigenvalues			Extraction sums of squared loadings		
	Total	% of variance	Cumulative %	Total	% of variance	Cumulative %
1	11.534	14.239	14.239	11.534	14.239	14.239
2	6.818	8.418	22.657	6.818	8.418	22.657
3	6.127	7.565	30.221	6.127	7.565	30.221
4	4.784	5.906	36.127	4.784	5.906	36.127
5	3.832	4.731	40.858	3.832	4.731	40.858
⋮	⋮	⋮	⋮	⋮	⋮	⋮
23	1.013	1.251	78.449	1.013	1.251	78.449
24	0.999	1.233	79.682			
⋮	⋮	⋮	⋮			
81	0.001	0.001	100.000			

**Table 4:** Confusion matrix.

Reported	Predicted	
	Fraud (1)	Nonfraud (0)
Fraud (1)	TP	FN
Nonfraud (0)	FP	TN

**Table 5:** Comparison of identification results by introduced text information.

Variable	Accuracy	Precision	Recall	*F*1-score	AUC
*F*	0.8447	0.8654	0.8333	0.8490	0.8452
*F* + *T*	0.8738	0.8571	0.9057	0.8807	0.8733

**Table 6:** The influence of each text index on the identification results.

Variable	Accuracy	Precision	Recall	*F*1-score	AUC
*F* + sentiment polarity	0.8544	0.8298	0.8478	0.8387	0.8567
*F* + emotional tone	0.8252	0.8200	0.8200	0.8200	0.8251
*F* + text readability	0.8641	0.8636	0.8261	0.8444	0.8604

**Table 7:** Analysis based on financial and nonfinancial variables.

Model	Accuracy	Precision	Recall	*F*1-score	AUC
RF	0.8155	0.8182	0.8333	0.8257	0.8146
AdaBoost	0.8155	0.8367	0.7885	0.8119	0.8158
GBDT	0.8058	0.8654	0.7895	0.8257	0.8057
Stacking	0.8447	0.8654	0.8333	0.8490	0.8452

**Table 8:** Introduction of text variables.

Model	Accuracy	Precision	Recall	*F*1-score	AUC
RF	0.8252	0.8541	0.7885	0.8200	0.8256
AdaBoost	0.8350	0.7679	0.9149	0.8350	0.8414
GBDT	0.8252	0.8333	0.8000	0.8163	0.8245
Stacking	0.8738	0.8571	0.9057	0.8807	0.8733

## Data Availability

The financial and nonfinancial data used to support the findings of this study have been deposited in the CSMAR repository (https://www.gtarsc.com/). The MD&A text data used to support the findings of this study are available from the corresponding author upon request.
